# Evaluation of adapted parent training for challenging behaviour in pre-school children with moderate to severe intellectual developmental disabilities: A randomised controlled trial

**DOI:** 10.1371/journal.pone.0306182

**Published:** 2024-08-13

**Authors:** Rachel Royston, Michael Absoud, Gareth Ambler, Jacqueline Barnes, Rachael Hunter, Marinos Kyriakopoulos, Tamara Ondruskova, Kate Oulton, Eleni Paliokosta, Monica Panca, Aditya Sharma, Vicky Slonim, Una Summerson, Alastair Sutcliffe, Megan Thomas, Chen Qu, Angela Hassiotis

**Affiliations:** 1 Division of Psychiatry, University College London, London, United Kingdom; 2 Evelina London Children’s Hospital, St Thomas’ Hospital, and King’s College London, London, United Kingdom; 3 Department of Statistical Science, University College London, London, United Kingdom; 4 Department of Psychological Sciences, Birkbeck University of London, London, United Kingdom; 5 Research Department of Primary Care and Population Health, Royal Free Medical School, London, United Kingdom; 6 1st Department of Psychiatry National and Kapodistrian University of Athens, Greece and South London and Maudsley NHS Foundation Trust and Department of Child and Adolescent Psychiatry, King’s College London, London, United Kingdom; 7 Great Ormond Street Hospital for Children NHS Foundation Trust, London, United Kingdom; 8 The Tavistock and Portman NHS Foundation Trust, Kentish Town Health Centre, London, United Kingdom; 9 Cumbria, Northumberland, Tyne & Wear NHS Foundation Trust & Newcastle University, Walkergate Park Centre for Neurorehabilitation and Neuropsychiatry, Newcastle, United Kingdom; 10 Contact, London, United Kingdom; 11 Institute of Child Health, University College London, London, United Kingdom; 12 Blackpool Teaching Hospitals NHS Foundation Trust, Blackpool, United Kingdom; Cardiff University, UNITED KINGDOM

## Abstract

**Objectives:**

There is limited evidence on the effectiveness of parenting interventions to improve disruptive behaviour in children with intellectual developmental disabilities. This clinical trial evaluated whether an adapted group parenting intervention for preschool children with intellectual developmental disabilities who display challenging behaviour is superior to treatment as usual in England.

**Study design:**

261 children aged 30–59 months with moderate to severe intellectual developmental disabilities and challenging behaviour were randomised to either the intervention (Stepping Stones Triple P) and treatment as usual or treatment as usual alone. The primary outcome was the parent-rated Child Behaviour Checklist at 52 weeks after randomisation. A health economic evaluation was also completed.

**Results:**

We found no significant difference between arms on the primary outcome (mean difference -4.23; 95% CI: -9.99 to 1.53; p = 0.147). However, a subgroup analysis suggests the intervention was effective for participants randomised before the COVID-19 pandemic (mean difference -7.12; 95% CI: -13.44 to -0.81; p = 0.046). Furthermore, a complier average causal effects analysis (mean difference -11.53; 95% CI: -26.97 to 3.91; p = 0.143) suggests the intervention requires participants to receive a sufficient intervention dose. The intervention generated statistically significant cost savings (-£1,057.88; 95% CI -£3,218.6 to -£46.67) but the mean point estimate in Quality Adjusted Life Years was similar in both groups.

**Conclusion:**

This study did not find an effect of the intervention on reducing challenging behaviour, but this may have been influenced by problems with engagement. The intervention could be considered by services as an early intervention if families are supported to attend, especially given its low cost.

## Introduction

Intellectual developmental disability (IDD) is a lifelong condition that affects an individual’s cognitive and adaptive functioning and manifests early in development [1]. There is significant evidence indicating that children with IDD display twice the rate of mental health comorbidities including challenging behaviour, than children without IDD [1, 2]. Challenging behaviour, which consists of disruptive behaviours and aggression towards peers or family, persists and contributes to lifelong poor health and social outcomes [3–5].

Available research indicates that group parent training is clinically effective in reducing challenging behaviour [6] and economic evaluations also indicate that such programmes can be cost-effective [7–10]. Early intervention appears to increase parent efficacy in managing the child’s behaviour and programmes targeting challenging behaviours in children without IDD may start in children as young as 18 months [11, 12].

National reports in the United Kingdom (UK) suggest that despite many government initiatives purporting to address the needs of children with IDD and of their families, timely access and delivery of preventive or treatment approaches remain out of reach [13]. Universal parenting programmes are often perceived as unsuitable to the specific needs of children with IDD and therefore are unlikely to be accessed by these families [14]. The National institute of Health and Care Excellence recognized the paucity of evidence for early interventions, especially for the group of children with more severe IDD, calling for further research into this clinically relevant topic area [14]. Whilst there is some evidence for the effectiveness of interventions which have been designed or adapted for children with IDD (e.g. Parent-Child Interaction Therapy, Incredible Years Parenting Training Programme) [15, 16], many of these studies are limited by small sample sizes, high attrition, limited generalisability and an absence of longer-term follow-up data [16, 17].

Studies outside of the UK have shown the acceptability and effectiveness of a well- known adapted universal programme called Group Stepping Stones Triple P (SSTP) [18] to reduce challenging behaviour and improve parenting styles [18–22]. SSTP is adapted from the universal Triple P (Positive Parenting Programme) for children with IDD, originating from Australia. The programme, delivered to a group of parents, covers strategies to reduce unwanted behaviours, maintain behavioural change, cultivate a positive relationship with the child and facilitate independent problem solving. However, most studies have been conducted within Australia [18] and there are some studies that have not found a positive effect of SSTP on child behaviour (e.g. a randomised controlled trial in the Netherlands [23]). Findings may be affected by different health and social care systems; hence this study aims to test SSTP as an early intervention for very young children with moderate to severe IDD in the UK context.

We conducted a randomised controlled trial to investigate whether level 4 SSTP in addition to treatment as usual (TAU) was clinically and cost effective in reducing parent reported challenging behaviour in children aged 30–59 months (aged approximately 2.5–5 years) with moderate-severe IDD at 52 weeks. Secondary hypotheses were that receiving SSTP compared to TAU alone would: 1) reduce challenging behaviour at 52 weeks in blind-rated observations and caregiver/teacher reports; 2) improve parental psychological wellbeing; 3) reduce parental stress; 4) increase parenting competence; and 5) be cost-effective.

## Materials and methods

The trial is described here using the CONSORT reporting guidelines [24]. The published protocol for the ‘Evaluation of Parent Intervention for Challenging Behaviour in Children with Intellectual Disabilities’ (EPICC-ID) trial [25] gives a fuller account of the methods and schedule of assessments. The funders report is also now available [26]. Trial registration number: https://clinicaltrials.gov NCT03086876.

### Study design and participants

The study was a pragmatic research assessor-blinded randomised controlled trial carried out in four sites in England (June 2017—December 2021). Participants were identified via the National Health Service Paediatric services or adjacent Participant Identification Centres, usually community paediatric teams, primary care, third sector parent organizations and by word of mouth. Eligible parent-child dyads were enrolled if: 1) parents were at least 18 years of age; and 2) the child was 30–59 months of age at identification and had moderate to severe IDD (General Adaptive Functioning score of 40–69) as assessed by the parent-reported Adaptive Behaviour Assessment System (ABAS)); and 3) displayed challenging behaviour for 2 months or longer (indicated through referral to services or due to reported parental concerns). Participants were excluded if: 1) the child had either mild, profound or no IDD on parent-reported ABAS; 2) the parent/carer had insufficient English; or 3) another sibling was taking part in the study. Parents provided written or audio-recorded verbal informed consent. The trial was approved by the London Camden & Kings Cross Research Ethics Committee (reference 17/LO/0659). A Trial Steering Committee and a Data Safety Monitoring Board oversaw study conduct.

### Intervention

Level 4 SSTP is a 9-week psycho-education programme that includes 6 face-to-face group sessions and 3 individual telephone or in-person contact with participants. Each group session lasts approximately 2.5 hours and individual sessions up to 30 minutes.

Therapists were eight professionals from social care, allied health professions and nursing backgrounds and were trained during a 3-day training course.

### Intervention fidelity

During the trial, therapist supervision was provided by programme developers and a trained co-applicant to support delivery fidelity and issues specific to trial conduct. Individual session checklists were completed by therapists and sessions were video recorded, with 10% (13) randomly selected and assessed by an external reviewer with expertise in SSTP. All scored sessions were rated highly for fidelity, ranging from 7 to 10 (M = 9.38, SD = 0.96) indicating high levels of treatment adherence [26]. Two sessions were rated as being of adequate quality; the remaining sessions were rated as good quality.

### Treatment as usual

TAU was defined as local access for families to interventions and therapies. Services included, but were not limited to, health visitors (approximately 4 visits in England), primary care engagement and advice, elements of early intervention provided by community paediatric services, and awareness training related to broadly defined IDD including autism spectrum disorders. A survey of the participating areas revealed that some other parenting programmes were available in each of the study areas (e.g., Early Bird parenting programme, The Incredible Years, The Family Links Course and universal Triple P) although none were received by the study participants whilst enrolled in the present trial.

### Outcomes

The primary endpoint was parent-reported challenging behaviour measured by the Child Behaviour Checklist (CBCL) at 52 weeks. The CBCL comprises 100 questions rated on a 3 point Likert scale [27]. The scale has been used to measure treatment efficacy in child populations. Secondary outcome measures were: other Child Behaviour Checklist Caregiver-Teacher Report Forms (C-TRF) [27]; observed challenging child behaviour based on 20 minutes of video recorded structured parent-child interaction tasks in the home scored using the Revised Family Observation Schedule [28]; non-psychotic and common psychiatric disorders in the parent measured by the General Health Questionnaire-12 [29]; parent stress measured by the Questionnaire on Resources and Stress short form [30]; frequency of difficult child behaviour during care-giving tasks measured by the Caregiving Problem Checklist-Difficult Child Behaviour [31]; Satisfaction and Efficacy as a parent measured by the Parenting Sense of Competence Scale [32]; and Client Satisfaction Questionnaire to examine parent acceptability of the intervention. Health and social care resource use was captured by the Child and Adolescent Service Use Schedule [33] and parent and child health related quality of life was measured by the EQ-5D-5L [34] and Pediatric Quality of Life Inventory (PedsQL) General Core Scales [35, 36] respectively.

All outcomes were assessed pre-randomisation, at 16 weeks and 52 weeks post-randomisation by trained researchers. Children were assessed at baseline with the ABAS [37] and the Mullen Scales of Early Learning [38]. Serious adverse events were monitored for both arms until the completion of follow-ups.

### Sample size

We required a sample size of 258 children (SSTP: 155 children, TAU: 103 children) to detect a low to moderate (standardised) effect size of 0.40 for the primary outcome at the 5% significance level, with 90% power. This is equivalent to detecting a clinically meaningful change in the mean raw scores between the two groups of 8 points, assuming a standard deviation of 20.

The calculation was based on an analysis using a baseline-adjusted mixed model, assuming a correlation of 0.5 between baseline and follow-up measurements and adjustment for therapist clustering (intra-class correlation of 0.05), with an average group size of seven as advised by the SSTP developers), and an anticipated drop-out of 10%.

### Randomisation and masking

Participants were randomly assigned into the intervention and control arms on a 3:2 ratio using randomly permuted blocks of varying block sizes and stratification by site and level of IDD (moderate and severe). The unequal randomisation ratio was a consequence of adjustment for therapist clustering. The process was aided by an online independent randomisation service with allocation concealment. The chief investigator and senior trial statistician were blinded to arm allocation as were the research assessors. In cases where allocation was inadvertently disclosed, other researchers blind to arm allocation carried out the assessments.

### COVID-19 impact

The study continued during the pandemic, with the last 12-month follow-up contact in December 2021. Research processes were adapted to facilitate verbal consenting (oral consent was introduced from March 2020 and recordings were saved into Data Safe Haven, a secure data platform), remote assessments, and online intervention delivery. Twenty SSTP groups were run face-to-face before the pandemic and five groups were run online once the pandemic started. The final online group combined participants from all study sites. During the pandemic, we were unable to carry out the Mullen Scales of Early Learning for 52 participants, nor the observations for 252 assessments (121 participants).

### Statistical methods

All statistical tests and confidence intervals were two-sided, and significance was set at 5%. The primary analysis was on an ‘intention to treat’ basis including all participants with available outcome data (CBCL) at 12 months. All analyses were performed using Stata version 17. Missing values in the outcomes were handled, where possible, using guidance from the corresponding outcome manual. For example, it is suggested that the CBCL score should not be calculated if there are more than 8 missing items; otherwise, missing items should be replaced with a 0 and the score calculated. The statistical analysis plan is available on request.

The primary outcome was analysed using a mixed model with fixed effects for randomisation group, CBCL total score at baseline, centre and level of IDD, and a random effect for therapist group (intervention arm only) [39]. We also analysed the externalising CBCL domain T-scores separately using the same approach. Several sensitivity analyses were performed for the primary analysis, including a per-protocol analysis, which excluded non-adhering participants (those attending less than a pre-specified number of sessions, i.e., at least 4 (out of 6) group sessions and 2 (out of 3) individual sessions) and a ‘Complier Average Causal Effects’ (CACE) analysis, which adjusted for intervention dose variability whilst maintaining randomisation balance; briefly, this analysis used a two-stage least squares approach to compare participants who received a sufficient dose of the intervention with TAU participants who would have received a sufficient dose if randomised to the SSTP arm [40]. Further sensitivity analyses used a mixed model to analyse both the 16- and 52-week CBCL outcomes simultaneously and used multiple imputation with chained equations to impute missing CBCL data. A pre-specified subgroup analysis was also performed to understand the effect of COVID-19 on this trial. Secondary outcomes were analysed using analogous regression methods to those described above. The presentation of all findings is in accordance with the latest CONSORT statement for pragmatic trials.

### Health economic analysis

The primary economic outcome measure was the Quality Adjusted Life Years (QALYs) derived from utility scores, obtained using the PedsQL scores. Mapped EQ-5D-Y utility scores algorithms [34] were used to provide an empirical basis for estimating health utilities. These were used to form QALYs over the 12-month period, adjusting for any imbalances in baseline scores [41]. Study records were used to track resources used in the delivery of the training course including preparation, therapist and trainer time, travel costs, attendance incentives and course materials to calculate the fixed cost of training. For the delivery of the intervention, the number of sessions and the time each therapist spent with a family was recorded as well as the cost of any materials provided to parents/caregivers. Health and social care resource use was costed using unit costs from the most recent Unit Costs of Health and Social Care [42]) and NHS reference costs. The costs of medications were estimated from the British National Formulary in 2019/2020 pounds sterling. Discounting was not applied, as trial follow-up did not exceed 52 weeks. Missing data was explored to determine its patterns, extent, and association with any participant characteristics.

The primary analysis included all participants using multiple imputation to predict missing costs and outcomes [43]. Cost and QALY data were combined to calculate an incremental cost-effectiveness ratio. Seemingly unrelated regression (SUR) [44] as used to account for correlation between costs and outcomes, with adjustment for baseline costs, utilities, CBCL, site and level of intellectual disability. The health economic analysis plan is also available on request.

## Results

**Fig 1:**
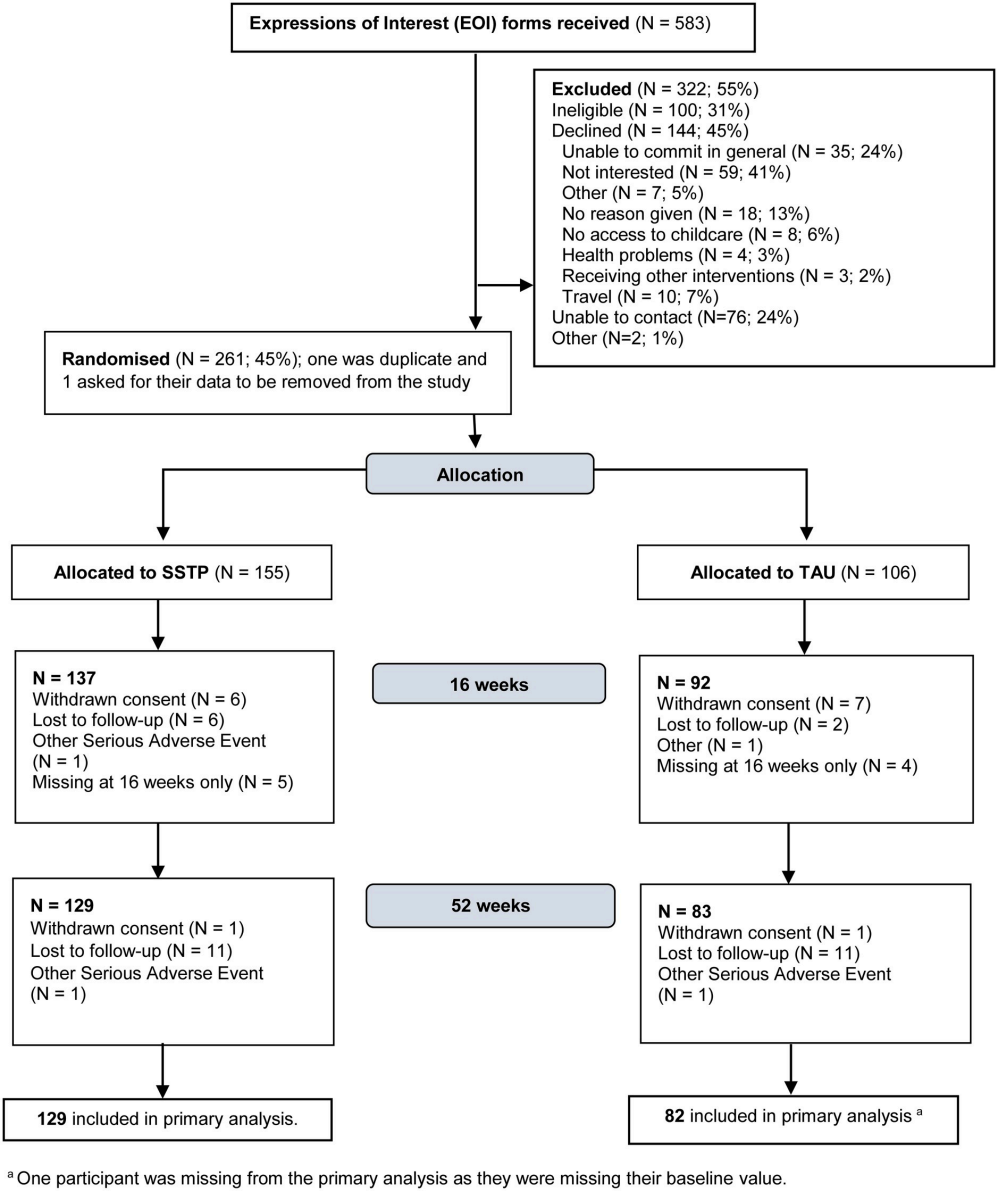
CONSORT flow diagram of recruitment.

In total, 261 participants were enrolled into the trial and randomised between September 2017 and December 2020 (SSTP: N = 155, TAU: N = 106). 51 participants were enrolled and randomised during the COVID-19 pandemic (16^th^ March 2020 – 17^th^ December 2020). Recruitment ended once the target sample size was reached. Overall, 219 assessments (baseline and follow-up) were carried out from that date to the last participant follow-up assessment in December 2021. 229 (88%) of 261 completed the 16-week assessments and 212 (81%) of 261 completed the 52-week assessments. Fig 1 shows a CONSORT flow diagram summarizing the flow of participants into the trial.

### Baseline characteristics

Child and parent baseline characteristics were balanced between the allocation arms (see details in Tables 1 and 2 by arm and for the whole sample). The participating children had mean (SD) age of 3.7 (1.0) years, 75% were male and 57% were reported by parents to be of White ethnicity. The participating parents had mean (SD) age of 34.4 (6.4) years at baseline and 62% self-identified as white.

**Table 1:** Child baseline characteristics.

	TAU	SSTP	Total
	(N = 106)	(N = 155)	(N = 261)
	Mean (SD);	Mean (SD);	Mean (SD);
	Median (IQR)	Median (IQR)	Median (IQR)
**Age (years)**	3.7 (0.9);	3.7 (1.0);	3.7 (1.0);
	3.8 (3.2,4.3)	3.9 (3.2,4.4)	3.9 (3.2,4.4)
**Mullen Scales of Early Learning scores**			
Visual Reception T Score^a^	25.9 (10.7)	25.1 (10.3)	25.4 (10.4)
Fine Motor T Score^b^	23.8 (8.2)	23.8 (9.3)	23.8 (8.8)
Receptive Language T score^a^	23.7 (8.0)	24.6 (8.9)	24.3 (8.5)
Expressive Language T Score^b^	24.3 (9.1)	24.1 (7.7)	24.2 (8.3)
Early Learning Composite Standard Score^c^	56.1 (11.7)	55.6 (13.1)	55.8 (12.5)
	**N (%)**	**N (%)**	**N (%)**
**Sex**			
Female	29 (27.4%)	37 (23.9%)	66 (25%)
Male	77 (72.6%)	118 (76.1%)	195 (75%)
**Ethnicity** ^d^			
Asian	11 (10.4%)	17 (11.0%)	28 (11%)
Black	16 (15.1%)	25 (16.1%)	41 (16%)
Mixed	10 (9.4%)	19 (12.3%)	29 (11%)
Other	7 (6.6%)	7 (4.5%)	14 (5%)
White	62 (58.5%)	87 (56.1%)	149 (57%)
**Severity of intellectual disabilities (based on ABAS)** ^e^			
Moderate	101 (95.3%)	151 (97.4%)	252 (97%)
Severe	5 (4.7%)	4 (2.6%)	9 (3%)
**Autism Spectrum Disorder as per participant clinical record**			
Yes	68 (64.2%)	90 (58.4%)	158 (61%)
No	38 (35.8%)	64 (41.6%)	102 (39%)
**Physical Health Problem**			
**No**	45 (42.5%)	76 (49.0%)	121 (46%)
**Yes** ^f^	61 (57.5%)	79 (51.0%)	140 (54%)
Mobility Difficulties	21 (34.4%)	31 (39.2%)	52 (37%)
Sensory Impairments	32 (52.5%)	41 (51.9%)	73 (52%)
Epilepsy	6 (9.8%)	5 (6.3%)	11 (8%)
Constipation	13 (21.3%)	26 (32.9%)	39 (28%)
**Education, Health, or Care Plan**			
Yes	36 (34.0%)	61 (39.4%)	97 (37%)
No	70 (66.0%)	94 (60.6%)	164 (63%)

**Note.** Categorical variables are summarised using frequencies (N) and percentages (%), while continuous variables are summarised using means, standard deviations (SD), medians and inter-quartile ranges (IQR). Summary statistics are presented for the total group and by allocation arm: TAU and SSTP

^a^ Mullen Scales of Early Learning scores based on the following pre-pandemic sample sizes: TAU (n = 69), SSTP (n = 99), total (n = 168)

^b^ Mullen Scales of Early Learning scores based on the following pre-pandemic sample sizes: TAU (n = 69), SSTP (n = 101), total (n = 170)

^c^ Mullen Scales of Early Learning scores based on the following pre-pandemic sample sizes: TAU (n = 68), SSTP (n = 98), total (n = 166)

^d^ Ethnicity as self-reported by parents

^e^ Since the start of the pandemic, we moved to remote assessments under which circumstances we were unable to administer the Mullen Scales of Early Learning, therefore we chose to report the ABAS results for all children

^f^ Children may have multiple physical health problems

**Table 2:** Parent baseline characteristics.

	TAU	SSTP	Total
	(N = 106)	(N = 155)	(N = 261)
	Mean (SD);	Mean (SD);	Mean (SD);
	Median (IQR)	Median (IQR)	Median (IQR)
**Age (years)**	34.8 (6.2);	34.0 (6.6);	34.4 (6.4);
	35(31,39)	33(29,38)	34(30,39)
	**N (%)**	**N (%)**	**N (%)**
**Respondent**			
Mother	94 (88.7%)	143 (92.3%)	237 (90.8%)
Father	11 (10.4%)	10 (6.5%)	21 (8.1%)
Other	1 (0.9%)	2 (1.3%)	3 (1.2%)
**Ethnicity** ^a^			
Asian	12 (11.3%)	17 (11.0%)	29 (11%)
Black	18 (17.0%)	29 (18.7%)	47 (18%)
Mixed	3 (2.8%)	6 (3.9%)	9 (3%)
Other	7 (6.6%)	8 (5.2%)	15 (6%)
White	66 (62.3%)	95 (61.3%)	161 (62%)
**Living Situation**			
Owned property	31 (29.2%)	29 (18.7%)	60 (23%)
Rented property	71 (67.0%)	123 (79.4%)	194 (74%)
Other	4 (3.8%)	3 (1.9%)	7 (3%)
**Employment Status**			
Unemployed/in education	6 (5.6%)	15 (9.7%)	21 (8%)
Part-time paid employment—<30 hrs/wk	31 (29.2%)	47 (30.3%)	78 (30%)
Full-time paid employment	14 (13.2%)	11 (7.1%)	25 (10%)
Looking after home and family	53 (50.0%)	77 (49.7%)	130 (50%)
Other	2 (1.9%)	5 (3.2%)	7 (3%)
**Relationship Status**			
Single	25 (23.6%)	52 (33.5%)	77 (30%)
Married/cohabiting	73 (68.9%)	92 (59.3%)	165 (64%)
Divorced/separated	8 (7.6%)	11 (7.1%)	19 (7%)
**Main Income** **(may be overlap between types)**			
Salary/Wage	58 (54.7%)	82 (52.9%)	140 (54%)
Family Support	11 (10.4%)	25 (16.1%)	36 (14%)
State benefit/Other	95 (89.6%)	144 (92.9%)	239 (91%)
**Mental ill-health problems**			
Yes	32 (30.2%)	41 (26.5%)	73 (28%)
No	74 (69.8%)	114 (73.5%)	188 (72%)
**Alcohol or drug abuse**			
Yes	0 (0%)	3 (1.9%)	3 (1%)
No	106 (100%)	152 (98.1%)	258 (99%)
**Family violence**			
Yes	7 (6.6%)	11 (7.1%)	18 (7%)
No	99 (93.4%)	144 (92.9%)	243 (93%)

Note. Categorical variables are summarised using frequencies (N) and percentages (%), while continuous variables are summarised using means, standard deviations (SD), medians and inter-quartile ranges (IQR). Summary statistics are presented for the total group and by allocation arm: treatment as usual (TAU) and intervention arm Stepping Stones Triple P (SSTP)

^a^ Ethnicity as self-reported by parents

### Delivery

Ninety-one (58.7%) participants randomised to SSTP attended at least one group session; of these the median (interquartile range) number of sessions attended was 5 (3 to 6). Sixty-six (42.6%) participants attended at least one individual session; of these the median (interquartile range) number of sessions attended was 3 (2 to 3). Fifty (32.3%) participants attended the pre-specified number of sessions, i.e., at least four (out of 6) group sessions and two (out of 3) individual sessions. There was a median of 47 days between the baseline assessment and start of therapy (interquartile range = 23 to 79 days). Thirteen individuals randomised to SSTP were invited to attend therapy but did not sign up.

### Analysis of the primary outcome

The primary outcome was available for 129/155 (83%) SSTP participants, and 83/106 (78%) TAU participants, although 82 were only included in the analysis due to one participant having missing baseline values. There were no differences in the baseline data for the participants with complete follow-up data at 52 weeks (n = 211) and those with missing data (n = 60). Table 3 shows the CBCL raw total scores, stratified by allocation arm at baseline, 16 and 52 weeks. At the primary endpoint of 52 weeks, the difference in CBCL score (intervention vs TAU) was -4.23 points (95% CI: -9.99 to 1.53; p-value = 0.147). At 16 weeks, the difference was -2.55 (95% CI: -7.63 to 2.53; p-value = 0.322) (see Table 3).

**Table 3:** Summary statistics for the primary and secondary outcomes, 16 and 52 weeks and estimate of intervention effect at follow-up.

	Allocation arm				Estimate of intervention effect Mean difference (95% CI)	p-value
	TAU (N = 106)		SSTP (N = 155)		SSTP vs TAU	
	N	Mean (SD)	N	Mean (SD)		
**Primary outcome**						
**Child Behaviour Checklist (CBCL) raw scores**						
Baseline	105	93.3 (28.3)	155	96.1 (24.8)		
16 weeks	92	91.6 (32.3)	137	91.5 (28.9)	-2.55 (-7.63, 2.53)	0.322
52 weeks	82	91.0 (30.1)	129	90.0 (31.2)	-4.23 (-9.99, 1.53)	0.147
**Secondary outcomes**						
**Child Behaviour Checklist (CBCL)–Externalising domain T-scores**						
Baseline	105	32.2 (9.4)	155	33.1 (8.5)		
Week 16	91	31.3 (10.8)	137	30.9 (9.9)	-1.21 (-2.97, 0.54)	0.174
Week 52	82	30.1 (10.1)	129	29.7 (10.4)	-1.59 (-3.58, 0.39)	0.114
**Child Behaviour Checklist Caregiver-Teacher Report Forms (C-TRF) raw scores**						
Baseline	52	70.1 (28.3)	90	69.6 (33.1)		
Week 16	28	64.9 (29.8)	47	67.4 (32.8)	6.17 (-7.05, 19.38)	0.351
Week 52	20	58.5 (27.8)	35	68.6 (28.5)	10.92 (-4.07, 25.91)	0.147
**General Health Questionnaire (GHQ-12**						
Baseline	104	14.8 (6.3)	150	15.1 (6.4)		
Week 16	84	13.2 (5.9)	128	13.0 (6.2)	-0.23 (-1.75, 1.29)	0.764
Week 52	72	13.2 (6.6)	110	13.2 (6.8)	-0.42 (-2.37, 1.52)	0.666
**Questionnaire on Resources and Stress (QRS-F short form)**						
Baseline	93	15.1 (3.8)	141	16.0 (4.1)		
Week 16	72	15.5 (4.5)	116	15.7 (4.2)	-0.38 (-1.40, 0.63)	0.456
Week 52	60	14.8 (4.3)	96	15.2 (3.9)	0.09 (-1.02, 1.20)	0.874
**Caregiving Problem Checklist-Difficult Child Behaviour**						
Baseline	100	33.5 (8.8)	144	34.1 (7.8)		
Week 16	80	32.4 (10.4)	120	33.5 (7.7)	0.09 (-2.08, 2.25)	0.937
Week 52	69	31.3 (9.8)	99	30.5 (9.5)	-2.05 (-4.72, 0.61)	0.129
**Parenting Sense of Competence Scale (PSOC)**						
Baseline	99	67.5 (11.2)	143	69.4 (11.3)		
Week 16	79	69.9 (11.4)	118	68.9 (10.8)	-1.66 (-4.14, 0.81)	0.186
Week 52	68	71.8 (12.9)	102	70.0 (10.0)	-2.20 (-5.29, 0.88)	0.160

**Note**. Continuous variables are summarised using means and standard deviations (SD). Summary statistics are presented for the total group and by allocation arm. Estimate of intervention effect for CBCL total score at 16 weeks: N (Total) = 228, N (TAU) = 91, N (SSTP) = 137. Estimate of intervention effect at 52 weeks: N (Total) = 211, N (TAU) = 82, N (SSTP) = 129. Analyses were adjusted for centre, baseline values of CBCL total and participant’s level of IDD.

### Sensitivity analyses

The per-protocol analysis excluded 82 participants from the SSTP arm as they had not received the intervention and estimates that CBCL mean score difference at 52 weeks was lower in the intervention arm by -10.77 (95% CI: -19.12 to -2.42, p = 0.014). When outcomes at 16- and 52-weeks were analysed together, CBCL mean score difference at 52 weeks was -4.49 (95% CI: -9.56 to 0.57, p = 0.082). The CACE analysis, which adjusts for intervention dose variability, estimates the CBCL mean score difference at 52 weeks was -11.53 (95% CI: -26.97 to 3.91, p = 0.143). When missing outcome values were imputed, CBCL mean score difference at 52 weeks was -4.85 (95% CI: -10.24 to 0.54, p = 0.078).

### COVID-19 impact on primary outcome

The parent-reported CBCL mean score difference between participants who were recruited before and after the COVID-19 pandemic were estimated as -7.12 (95% CI: -13.44 to -0.81) and 7.61 (95% CI: -5.43 to 20.64) respectively (p = 0.046). This suggests the intervention may have been effective pre-pandemic, however the mean score differences were reduced once the pandemic began (see S1 Table).

### Analysis of secondary outcomes at 16 and 52 weeks

The non-significant reduction in the externalising domain CBCL t-scores in the SSTP arm at 16 weeks (-1.21, 95% CI -2.97 to 0.54, p = 0.174) remained at 52 weeks (-1.59, 95% CI -3.58 to 0.39, p = 0.114) (Table 3). We did not find any statistically significant differences at either assessment points post-randomisation in any of the secondary outcomes (see Table 3). The video-recorded child-parent interaction task (collected pre-pandemic), indicated child negative behaviours decreased at 52 weeks in both trial arms. There was no significant effect of arm allocation on the duration of parental display of positive and negative behaviours towards the child (see Table 4). The videos were rated by an experienced developmental psychologist blinded to allocation.

**Table 4:** Parent-child interaction scores at baseline, 16 and 52 weeks.

	Allocation Arm					
	TAU (N = 106)			SSTP (N = 155)		
	N	Mean (SD)	Range	N	Mean (SD)	Range
**Child Behaviours**						
**Positive behaviours**						
**Appropriate verbal activity**						
Baseline	77	7.3 (8.8)	0 to 33	102	9.1 (10.2)	0 to 36
Week 16	54	9.6 (9.5)	0 to 32	75	10.1 (10.7)	0 to 36
Week 52	35	12.2 (11.1)	0 to 29	45	12.6 (11.1)	0 to 36
**Engaged Activity**						
Baseline	77	19.4 (9.1)	1 to 39	102	17.0 (9.6)	0 to 38
Week 16	54	19.8 (8.5)	2 to 37	75	18.5 (9.0)	0 to 37
Week 52	35	17.0 (12.3)	1 to 40	45	17.3 (10.1)	1 to 38
**Negative behaviours**						
**Non-Compliance**						
Baseline	77	10.3 (6.4)	0 to 28	102	10.5 (6.6)	0 to 30
Week 16	54	8.0 (5.6)	0 to 19	75	9.2 (7.3)	0 to 32
Week 52	35	6.4 (5.5)	0 to 23	45	6.2 (5.2)	0 to 20
**Complaint**						
Baseline	77	3.6 (5.4)	0 to 29	102	4.3 (6.9)	0 to 40
Week 16	54	2.9 (3.6)	0 to 15	75	3.4 (5.4)	0 to 28
Week 52	35	2.9 (3.9)	0 to 16	45	1.9 (3.1)	0 to 16
**Physical Negative**						
Baseline	77	1.2 (2.8)	0 to 16	102	1.3 (2.4)	0 to 13
Week 16	54	1.1 (2.3)	0 to 9	75	0.9 (2.0)	0 to 12
Week 52	35	1.3 (2.2)	0 to 9	45	0.9 (2.1)	0 to 12
**Oppositional**						
Baseline	77	1.0 (1.7)	0 to 6	102	1.2 (2.0)	0 to 9
Week 16	54	0.9 (2.1)	0 to 10	75	0.5 (1.3)	0 to 7
Week 52	35	1.5 (2.7)	0 to 12	45	0.8 (1.8)	0 to 10
**Total negative behaviour** *						
Baseline	77	32.6 (20.3)	0 to 87.5	102	34.3 (21.1)	0 to 100
Week 16	54	26.3 (17.7)	0 to 65	75	28.3 (20.2)	0 to 80
Week 52	35	26.9 (20.0)	0 to 75	45	24.0 (18.2)	0 to 70
**Parent Behaviours**						
**Positive behaviours**						
**Praise**						
Baseline	77	5.7 (3.8)	0 to 14	102	5.0 (3.9)	0 to 21
Week 16	54	6.3 (3.7)	0 to 19	75	6.5 (4.5)	0 to 19
Week 52	35	7.5 (4.5)	0 to 20	45	5.0 (4.6)	0 to 18
**Contact Positive**						
Baseline	77	9.7 (7.4)	0 to 29	102	8.8 (7.7)	0 to 32
Week 16	54	8.6 (8.3)	0 to 29	75	9.8 (8.4)	0 to 38
Week 52	35	8.7 (8.3)	0 to 30	45	7.7 (6.2)	0 to 23
**Instruction**						
Baseline	77	25.7 (6.0)	10 to 38	102	25.3 (5.5)	6 to 39
Week 16	54	24.3 (4.7)	14 to 36	75	25.7 (4.3)	14 to 37
Week 52	35	25.5 (5.0)	14 to 36	45	23.6 (5.5)	9 to 36
**Social Attention**						
Baseline	77	6.4 (4.8)	0 to 23	102	6.9 (4.4)	0 to 23
Week 16	54	7.3 (4.6)	0 to 16	75	5.6 (3.8)	0 to 19
Week 52	35	7.2 (4.8)	0 to 16	45	7.2 (5.1)	0 to 19
**Affection**						
Baseline	77	8.8 (4.6)	1 to 18	102	7.8 (4.7)	0 to 21
Week 16	54	9.7 (4.9)	1 to 25	75	9.8 (4.9)	0 to 21
Week 52	35	11.1 (5.5)	3 to 22	45	8.0 (5.9)	1 to 26
**Negative behaviours**						
**Contact Negative**						
Baseline	77	0.0 (0.3)	0 to 2	102	0.1 (0.7)	0 to 6
Week 16	54	0.0 (0.1)	0 to 1	75	0.1 (0.7)	0 to 5
Week 52	35	0.1 (0.3)	0 to 2	45	0.1 (0.5)	0 to 3
**Instruction Negative**						
Baseline	77	0.1 (0.6)	0 to 5	102	0.3 (1.2)	0 to 9
Week 16	54	0.1 (0.5)	0 to 3	75	0.1 (0.4)	0 to 2
Week 52	35	0.2 (0.6)	0 to 3	45	0.0 (0.0)	0 to 0
**Social Attention Negative**						
Baseline	77	0.0 (0.0)	0 to 0	102	0.0 (0.1)	0 to 1
Week 16	54	0.0 (0.2)	0 to 1	75	0.0 (0.1)	0 to 1
Week 52	35	0.3 (1.9)	0 to 11	45	0.0 (0.0)	0 to 0
**Number of intervals**						
Baseline	77	39.6 (1.9)	26 to 40	102	39.8 (1.2)	33 to 40
Week 16	54	39.9 (0.8)	34 to 40	75	39.9 (0.7)	35 to 40
Week 52	35	39.9 (0.3)	38 to 40	45	39.2 (2.8)	26 to 40

*Notes*. Continuous variables are summarised using means, standard deviations (SD) and ranges.

Summary statistics are presented by allocation arm:

treatment as usual (TAU) and intervention arm Stepping Stones Triple P (SSTP)

*: indicated by the percentage of 30 second observation intervals where at least one (often more than one) of the negative behaviours had occurred (Non-Compliance, Complaint, Physical Negative, Oppositional, Interrupt).

### Cost effectiveness

We found that training in level 4 SSTP costs £26 per participant. From a health and social care perspective, SSTP dominates TAU with a mean cost saving of -£1,057.88 per participant (95% CI -£3,218.6 to -£46.67) and a mean QALY difference of 0.005 (95% CI -0.023 to 0.051) (see S2 and S3 Tables). Using NICE’s accepted cost-effectiveness thresholds, there is an 89% probability that SSTP is cost-effective compared to TAU at a threshold of £20,000 and £30,000 per QALY gained.

### Adverse events

20 serious adverse events were reported (SSTP: N = 12; TAU: N = 8). Of these, 13 related to children (1 event per child) and 7 to parents. Three of these cases were related to the child being removed from the parent’s care. None of the events were deemed to be related to the intervention.

## Discussion

We did not find evidence that Level 4 SSTP added to TAU as delivered in this trial was superior to TAU at 52 weeks post-randomisation. However, additional analyses based on those who received at least half of the group sessions indicated that the magnitude of change was greater than those who did not and particularly in participants recruited pre-pandemic. The externalising CBCL domain T-scores show that the children were within the clinically significant range throughout the trial (>64). Regarding the secondary outcome of other carer-reported child behaviours, measured by the C-TRF, we found a non-statistically significant increase in child behaviours at both 16 and 52 weeks. Although this appears to be counterintuitive given that parent-reported child behaviours decreased, it may reflect both the remitting-relapsing nature of these behaviours as well as that they may alter in different settings, e.g., home or school.

The primary economic evaluation indicates there is a high probability that Level 4 SSTP is cost-effective compared to TAU. The cost of delivering training for the intervention was relatively low and was accompanied by higher QALYs in those who received SSTP compared to TAU only. Non-significant differences in service use between groups were found, indicating the ongoing increased needs of those families. Therefore, a rollout of an alternative medium intensity parenting programme such as SSTP combining both in group and individual sessions could be achievable within existing services. Funding decisions though may be dependent on whether challenging behaviour is prioritised for prevention and intervention within a host of other clinical considerations at local and national levels.

### Findings in context

A systematic review and meta-analysis [45] found SSTP to be effective however most included studies were conducted in Australia, limiting the generalisability of findings to other countries with different health and care systems. A recently updated meta-analysis of international trials of any level of SSTP included 16 studies and 900 participants, though none from the UK [18]. The authors found significant effect sizes (range 0.46–0.77) of SSTP for parent-reported child behaviours and other measures of parental efficacy. Although overall the studies were judged as low risk of bias, a third did not use intention to treat analysis and therefore, may have also contributed to a more positive result than would be warranted otherwise. The meta-analysis also found a significant effect of SSTP in reducing directly observed child negative behaviours, with no effects on the parents’ behaviour. This is similar to the findings in this study, although negative observed behaviours also decreased for the TAU group. This may be related to natural changes in behaviours over time, or it may reflect a regression to the mean and requires further investigation in future work.

The existing evidence base mostly explores broader age ranges of children [45] and it may be that the families of pre-school children in the UK have too complex needs and do not have the resources to prioritise early intervention for challenging behaviour. Similar challenges with delivery and attendance to groups were also reported in another UK based trial for pre-school children for the Incredible Years parent intervention, with only half of parents attending over 50% of sessions [46].

During the pandemic, parents in this trial reported increased stress, a deterioration in their own mental health and a cessation of support from educational, social and healthcare services [47]. This may have affected their ability to engage successfully with the intervention and they may have also found it harder to engage during online delivery, particularly whilst having their children at home. Many parents also found it challenging to transition to and utilise telehealth methods during this time [47]. The cessation of support services would likely have negatively impacted both groups. Future research is required to explore the most effective modes of intervention delivery for this population.

Regarding the costs of parent training interventions, most of the evidence is on children with autism and from studies carried out outside the UK. The NICE guideline 11 [14], found low-quality evidence that interventions specifically for challenging behaviour in children and young people with IDD is potentially cost effective, especially for cases of high levels of behaviour severity. Our trial is the only one to include a health economic evaluation relevant to the British National Health System and specifically for this patient population. From a public health perspective, a state-wide implementation of any level of SSTP as preferred by Australian participants showed that SSTP was accessed by 4 times as many families as the proportion who receive care from statutory services (38% vs 10%) and that it was cost effective with savings of $574 (AUS) per family per annum [48].

### Strengths and limitations

To our knowledge, this is the first pragmatic randomised controlled trial of an intervention to reduce challenging behaviour in pre-school children with moderate-severe IDD in the UK. This is a group that arguably presents with the highest range of challenging behaviour but also receives fewer targeted interventions compared with other patient groups [49]. The study also benefits from the first cost effectiveness analysis of a complex early intervention in this population, making it a significant contribution to the scientific literature and of benefit to commissioners of services and to policy makers. A significant proportion of participants (approximately 40%) were from minority ethnic groups which indicates the sample was representative of the population of families of children with disabilities referred to community paediatric services in England.

However, the study also has limitations. The main threat to the trial validity is the higher attrition than that included in the sample size estimation, though overall the study retained power as shown in the post-hoc analysis (power reduced from 90% to 89%). Further, there was lower than anticipated attendance to group and individual sessions. Results of the per protocol and CACE analyses indicate that dose is likely to be central to achieving the intervention effect though they must be interpreted with caution, given the underlying methodological assumptions of the models. We followed all necessary protocols pertaining to participant and researcher safety as were available during the pandemic. Further research is needed to explore the implementation of parenting groups for behavioural problems in this population, as well pathways to increase engagement and intervention dosage.

In addition, the removal of the Mullen Scales of Early Learning measure meant reports on child functioning relied predominantly on the parent-reported measure (ABAS), and this may not provide an accurate estimation of IDD severity. It is possible that although all children were within clinical range for externalising disorders, the improvement was more incremental than anticipated. The pandemic also made it much harder to identify other caregivers to complete the C-TRF, as families were unable to meet, and schools and nurseries were closed. Nevertheless, we were able to carry on with the study without having to pause and believe that we mitigated most challenges appropriately [50].

## Conclusion

Although the study did not show superiority of SSTP over TAU in England, there are indications the intervention may be beneficial under certain conditions, and highlights the importance of format of delivery, and implementation challenges. The intervention could be rolled out within UK services, although providing additional support to promote adherence will be crucial to facilitate uptake and positive clinical outcomes.

## Supporting information

S1 Table Summary statistics and results for the primary outcome pre and during the COVID-19 pandemic. (DOCX)

S2 Table Health and social care costs of participants in the EPICC-ID trial. (DOCX)

S3 Table Utilities and quality adjusted life years. (DOCX)

S1 Checklist Reporting checklist for randomised trial. (DOCX)

S2 Checklist *PLOS ONE* clinical studies checklist. (DOCX)

## Abbreviations

ABAS: Adaptive Behaviour Assessment System
CACE: Complier average causal effects
CBCL: Child Behaviour Checklist
C-TRF: Child Behaviour Checklist Caregiver-Teacher Report Forms
IDD: Intellectual Developmental Disabilities
PedsQL: Pediatric Quality of Life Inventory
QALY: Quality Adjusted Life Years
SSTP: Stepping Stones Triple P
TAU: Treatment as Usual
UK: United Kingdom
