# Supplementary material for: Evaluation of adapted parent training for challenging behaviour in pre-school children with moderate to severe intellectual developmental disabilities: A randomised controlled trial
Source: PLoS One. 2024 Aug 13;19(8):e0306182. doi: 10.1371/journal.pone.0306182 (PMC11321573; doi:10.1371/journal.pone.0306182)
Supplement: S1 Table — (DOCX) [file pone.0306182.s001.docx]

**S1 Table.** Summary statistics and results for the primary outcome pre and during the COVID-19 pandemic

|  | Allocation Arm | | | |  | |
| --- | --- | --- | --- | --- | --- | --- |
|  | **TAU (N = 106)** | | **SSTP (N = 155)** | | **SSTP vs TAU** | |
|  | **N** | **Mean (SD)** | **N** | **Mean (SD)** | **Mean**  **difference (95% CI)** | **P-value** |
| COVID-19 consideration - CBCL at 52 weeks | | | | | | |
| Baseline |  |  |  |  |  |  |
| Before 16 March 2020 | 86 | 91.7 (28.3) | 123 | 94.4 (24.8) |  |  |
| After 16 March 2020 | 19 | 100.8 (27.9) | 32 | 102.7 (23.8) |  |  |
| Week 16 |  |  |  |  |  |  |
| Before 16 March 2020 | 75 | 90.2 (33.3) | 110 | 88.4 (28.2) |  |  |
| After 16 March 2020 | 17 | 97.5 (27.8) | 27 | 104.3 (28.3) |  |  |
| Week 52 |  |  |  |  |  |  |
| Before 16 March 2020 | 68 | 91.2 (31.3) | 102 | 87.1 (30.1) | -7.12 (-13.44, -0.81) | 0.046 |
