# Supplementary material for: Evaluation of adapted parent training for challenging behaviour in pre-school children with moderate to severe intellectual developmental disabilities: A randomised controlled trial
Source: PLoS One. 2024 Aug 13;19(8):e0306182. doi: 10.1371/journal.pone.0306182 (PMC11321573; doi:10.1371/journal.pone.0306182)
Supplement: S2 Table — (DOCX) [file pone.0306182.s002.docx]

**S2 Table.** Health and social care costs of participants in the EPICC-ID trial

|  |  | **SSTP** | | | **TAU** | | |  |  |  |  |
| --- | --- | --- | --- | --- | --- | --- | --- | --- | --- | --- | --- |
|  |  | **N** | **Mean** | **SD** | **N** | **Mean** | **SD** | **Adjusted* mean difference** | **p-value** | **95% CI (Lower limit)** | **95% CI (Upper limit)** |
| **Hospital Services Costs** | | | |  |  |  |  |  |  |  |  |
| **Inpatient stays** | Baseline | 155 | 584 | 5005 | 106 | 336 | 1763 |  |  |  |  |
|  | Week 16 | 137 | 118 | 709 | 92 | 195 | 997 |  |  |  |  |
|  | Week 52 | 129 | 29 | 232 | 83 | 699 | 4842 |  |  |  |  |
|  | Total cost | 124 | 844 | 5808 | 79 | 1413 | 6637 | -903.45 | 0.057 | -1833.80 | 26.90 |
|  |  |  |  |  |  |  |  |  |  |  |  |
| **Outpatient appointments** | Baseline | 155 | 348 | 559 | 106 | 423 | 848 |  |  |  |  |
|  | Week 16 | 137 | 182 | 344 | 92 | 242 | 498 |  |  |  |  |
|  | Week 52 | 129 | 174 | 424 | 83 | 280 | 589 |  |  |  |  |
|  | Total cost | 124 | 667 | 1018 | 79 | 995 | 1716 | -101.58 | 0.247 | -274.10 | 70.94 |
|  |  |  |  |  |  |  |  |  |  |  |  |
| **Accident and Emergency Attendances** | Baseline | 155 | 72 | 168 | 106 | 117 | 292 |  |  |  |  |
|  | Week 16 | 137 | 41 | 119 | 92 | 61 | 141 |  |  |  |  |
|  | Week 52 | 129 | 49 | 251 | 83 | 64 | 210 |  |  |  |  |
|  | Total cost | 124 | 171 | 464 | 79 | 254 | 567 | 8.07 | 0.842 | -71.60 | 87.74 |
| **Community Services Costs** | | | |  |  |  |  |  |  |  |  |
| **General Practitioner** | Baseline | 155 | 57 | 99 | 106 | 72 | 122 |  |  |  |  |
|  | Week 16 | 137 | 37 | 137 | 91 | 36 | 69 |  |  |  |  |
|  | Week 52 | 129 | 24 | 44 | 83 | 42 | 95 |  |  |  |  |
|  | Total cost | 124 | 118 | 208 | 78 | 161 | 222 | -15.16 | 0.474 | -56.81 | 26.49 |
|  |  |  |  |  |  |  |  |  |  |  |  |
| **Practice nurse** | Baseline | 155 | 12 | 35 | 106 | 16 | 40 |  |  |  |  |
|  | Week 16 | 137 | 9 | 42 | 92 | 5 | 17 |  |  |  |  |
|  | Week 52 | 129 | 5 | 15 | 83 | 10 | 30 |  |  |  |  |
|  | Total cost | 124 | 26 | 72 | 79 | 33 | 62 | -1.60 | 0.785 | -13.16 | 9.96 |
|  |  |  |  |  |  |  |  |  |  |  |  |
| **Health visitor** | Baseline | 155 | 72 | 160 | 106 | 60 | 117 |  |  |  |  |
|  | Week 16 | 137 | 24 | 92 | 92 | 46 | 107 |  |  |  |  |
|  | Week 52 | 129 | 8 | 29 | 83 | 24 | 74 |  |  |  |  |
|  | Total cost | 124 | 97 | 209 | 79 | 133 | 205 | -53.08 | 0.001 | -85.37 | -20.79 |
|  |  |  |  |  |  |  |  |  |  |  |  |
| **Community paediatrician** | Baseline | 155 | 250 | 350 | 106 | 227 | 285 |  |  |  |  |
|  | Week 16 | 137 | 108 | 192 | 91 | 119 | 238 |  |  |  |  |
|  | Week 52 | 129 | 94 | 185 | 83 | 147 | 309 |  |  |  |  |
|  | Total cost | 124 | 480 | 559 | 78 | 506 | 545 | -72.84 | 0.139 | -169.54 | 23.85 |
|  |  |  |  |  |  |  |  |  |  |  |  |
| **Social worker** | Baseline | 155 | 49 | 197 | 106 | 36 | 131 |  |  |  |  |
|  | Week 16 | 137 | 42 | 284 | 92 | 43 | 221 |  |  |  |  |
|  | Week 52 | 129 | 85 | 517 | 83 | 24 | 82 |  |  |  |  |
|  | Total cost | 124 | 175 | 819 | 79 | 109 | 322 | 88.87 | 0.352 | -99.15 | 276.89 |
|  |  |  |  |  |  |  |  |  |  |  |  |
| **Educational psychologist** | Baseline | 155 | 47 | 201 | 106 | 26 | 67 |  |  |  |  |
|  | Week 16 | 137 | 15 | 40 | 91 | 21 | 64 |  |  |  |  |
|  | Week 52 | 129 | 18 | 46 | 83 | 39 | 165 |  |  |  |  |
|  | Total cost | 124 | 80 | 230 | 78 | 98 | 207 | -32.63 | 0.091 | -70.47 | 5.22 |
|  |  |  |  |  |  |  |  |  |  |  |  |
| **Special educational need co-ordinator/Early years co-ordinator** | Baseline | 155 | 38 | 105 | 106 | 33 | 98 |  |  |  |  |
|  | Week 16 | 137 | 22 | 124 | 91 | 13 | 27 |  |  |  |  |
|  | Week 52 | 129 | 17 | 62 | 83 | 17 | 46 |  |  |  |  |
|  | Total cost | 124 | 81 | 187 | 78 | 61 | 112 | 13.98 | 0.430 | -20.89 | 48.85 |
|  |  |  |  |  |  |  |  |  |  |  |  |
| **Any parental group** | Baseline | 155 | 34 | 91 | 106 | 16 | 67 |  |  |  |  |
|  | Week 16 | 137 | 51 | 89 | 92 | 13 | 50 |  |  |  |  |
|  | Week 52 | 128 | 17 | 51 | 83 | 13 | 66 |  |  |  |  |
|  | Total cost | 123 | 100 | 129 | 79 | 47 | 120 | 38.97 | 0.013 | 8.48 | 69.47 |
|  |  |  |  |  |  |  |  |  |  |  |  |
| **Play therapist** | Baseline | 155 | 26 | 183 | 106 | 15 | 89 |  |  |  |  |
|  | Week 16 | 137 | 8 | 48 | 92 | 13 | 67 |  |  |  |  |
|  | Week 52 | 129 | 1 | 7 | 83 | 13 | 73 |  |  |  |  |
|  | Total cost | 124 | 33 | 197 | 79 | 43 | 163 | -22.20 | 0.087 | -47.65 | 3.25 |
|  |  |  |  |  |  |  |  |  |  |  |  |
| **Art/music/drama therapist** | Baseline | 155 | 6 | 46 | 106 | 7 | 42 |  |  |  |  |
|  | Week 16 | 137 | 2 | 15 | 92 | 3 | 17 |  |  |  |  |
|  | Week 52 | 129 | 2 | 13 | 83 | 6 | 43 |  |  |  |  |
|  | Total cost | 124 | 8 | 51 | 79 | 14 | 68 | -6.54 | 0.233 | -17.33 | 4.25 |
|  |  |  |  |  |  |  |  |  |  |  |  |
| **Occupational therapist** | Baseline | 155 | 175 | 484 | 106 | 189 | 623 |  |  |  |  |
|  | Week 16 | 137 | 79 | 305 | 92 | 133 | 512 |  |  |  |  |
|  | Week 52 | 129 | 195 | 667 | 83 | 289 | 787 |  |  |  |  |
|  | Total cost | 124 | 450 | 1352 | 79 | 673 | 1637 | -88.81 | 0.436 | -313.21 | 135.58 |
|  |  |  |  |  |  |  |  |  |  |  |  |
| **Speech and language therapist** | Baseline | 155 | 407 | 729 | 106 | 459 | 924 |  |  |  |  |
|  | Week 16 | 137 | 235 | 556 | 92 | 302 | 806 |  |  |  |  |
|  | Week 52 | 129 | 363 | 1203 | 83 | 429 | 874 |  |  |  |  |
|  | Total cost | 124 | 1008 | 1889 | 79 | 1272 | 2250 | -50.78 | 0.812 | -471.72 | 370.16 |
|  |  |  |  |  |  |  |  |  |  |  |  |
| **Physiotherapist** | Baseline | 155 | 54 | 166 | 106 | 63 | 312 |  |  |  |  |
|  | Week 16 | 137 | 23 | 110 | 92 | 29 | 99 |  |  |  |  |
|  | Week 52 | 129 | 48 | 216 | 83 | 94 | 406 |  |  |  |  |
|  | Total cost | 124 | 114 | 421 | 79 | 209 | 790 | -4.78 | 0.886 | -70.68 | 61.11 |
|  |  |  |  |  |  |  |  |  |  |  |  |
| **Dietician/**  **Nutritionist** | Baseline | 155 | 16 | 56 | 106 | 34 | 142 |  |  |  |  |
|  | Week 16 | 137 | 11 | 38 | 92 | 13 | 47 |  |  |  |  |
|  | Week 52 | 129 | 6 | 35 | 83 | 5 | 22 |  |  |  |  |
|  | Total cost | 124 | 27 | 73 | 79 | 59 | 181 | 0.93 | 0.912 | -15.49 | 17.34 |
|  |  |  |  |  |  |  |  |  |  |  |  |
| **Osteopath** | Baseline | 155 | 0.3 | 3 | 106 | 2 | 12 |  |  |  |  |
|  | Week 16 | 137 | 0.3 | 3 | 92 | 0 | 0 |  |  |  |  |
|  | Week 52 | 129 | 0 | 0 | 83 | 0 | 4 |  |  |  |  |
|  | Total cost | 124 | 0.3 | 4 | 79 | 3 | 16 | 0.31 | 0.554 | -0.72 | 1.34 |
|  |  |  |  |  |  |  |  |  |  |  |  |
| **Alternative therapies** | Baseline | 154 | 0.6 | 7 | 104 | 23 | 168 |  |  |  |  |
|  | Week 16 | 134 | 0.4 | 4 | 88 | 119 | 1105 |  |  |  |  |
|  | Week 52 | 129 | 243 | 2679 | 80 | 5 | 40 |  |  |  |  |
|  | Total cost | 121 | 254 | 2765 | 71 | 41 | 258 | 251.63 | 0.470 | -434.71 | 937.97 |
|  |  |  |  |  |  |  |  |  |  |  |  |
| **Equipment** | Baseline | 155 | 113 | 674 | 106 | 70 | 268 |  |  |  |  |
|  | Week 16 | 137 | 100 | 666 | 92 | 36 | 206 |  |  |  |  |
|  | Week 52 | 129 | 33 | 173 | 83 | 34 | 129 |  |  |  |  |
|  | Total cost | 124 | 284 | 1377 | 79 | 112 | 306 | 39.20 | 0.561 | -93.67 | 172.06 |
| **Prescriptions** | Baseline | 155 | 61 | 148 | 106 | 96 | 413 |  |  |  |  |
|  | Week 16 | 137 | 38 | 98 | 92 | 46 | 148 |  |  |  |  |
|  | Week 52 | 129 | 54 | 144 | 83 | 106 | 381 |  |  |  |  |
|  | Total cost | 124 | 154 | 302 | 79 | 268 | 992 | -13.27 | 0.564 | -58.57 | 32.04 |
| **Out-of-Pocket Expenditure** | | | |  |  |  |  |  |  |  |  |
| **Equipment** | Baseline | 155 | 801 | 8032 | 106 | 106 | 232 |  |  |  |  |
|  | Week 16 | 137 | 65 | 286 | 92 | 53 | 140 |  |  |  |  |
|  | Week 52 | 129 | 792 | 5584 | 83 | 261 | 1646 |  |  |  |  |
|  | Total cost | 124 | 1006 | 5712 | 79 | 415 | 1698 | 497.23 | 0.468 | -851.42 | 1845.88 |
|  |  |  |  |  |  |  |  |  |  |  |  |
| **Supplements** | Baseline | 155 | 17 | 30 | 106 | 16 | 33 |  |  |  |  |
|  | Week 16 | 137 | 11 | 26 | 92 | 13 | 64 |  |  |  |  |
|  | Week 52 | 129 | 17 | 36 | 83 | 10 | 28 |  |  |  |  |
|  | Total cost | 124 | 48 | 63 | 79 | 41 | 99 | 1.28 | 0.878 | -15.15 | 17.71 |

*Notes.* Adjusted for baseline costs, site and level of IDD; Summary statistics presented as means and standard deviations (SD).
