# Supplementary material for: Evaluation of adapted parent training for challenging behaviour in pre-school children with moderate to severe intellectual developmental disabilities: A randomised controlled trial
Source: PLoS One. 2024 Aug 13;19(8):e0306182. doi: 10.1371/journal.pone.0306182 (PMC11321573; doi:10.1371/journal.pone.0306182)
Supplement: S3 Table — (DOCX) [file pone.0306182.s003.docx]

**S3 Table.** Utilities and quality adjusted life years

|  |  | **SSTP** | | | **TAU** | | |  |  |  |  |
| --- | --- | --- | --- | --- | --- | --- | --- | --- | --- | --- | --- |
|  |  | **N** | **Mean** | **SD** | **N** | **Mean** | **SD** | **Adjusted* mean difference** | **p- value** | **95% CI (Lower limit)** | **95% CI (Upper limit)** |
| **Child** | Baseline | 140 | 0.637 | 0.145 | 98 | 0.615 | 0.157 |  |  |  |  |
|  | Week 16 | 119 | 0.643 | 0.174 | 80 | 0.626 | 0.154 |  |  |  |  |
|  | Week 52 | 109 | 0.641 | 0.155 | 70 | 0.613 | 0.152 |  |  |  |  |
|  | QALYs | 89 | 0.653 | 0.138 | 59 | 0.609 | 0.140 | -0.002 | 0.930 | -0.038 | 0.035 |
|  |  |  |  |  |  |  |  |  |  |  |  |
| **Parent** | Baseline | 152 | 0.799 | 0.189 | 106 | 0.799 | 0.211 |  |  |  |  |
|  | Week 16 | 130 | 0.810 | 0.216 | 86 | 0.811 | 0.180 |  |  |  |  |
|  | Week 52 | 116 | 0.827 | 0.203 | 75 | 0.826 | 0.194 |  |  |  |  |
|  | QALYs | 108 | 0.820 | 0.171 | 67 | 0.823 | 0.148 | 0.007 | 0.718 | -0.032 | 0.046 |

*Notes.* Adjusted for baseline utility values, site, and level of IDD; Summary statistics presented as means and standard deviations (SD)
